# Marginal notes, September 2024: in the antimicrobial resistance hot seat

**DOI:** 10.1099/jmm.0.001906

**Published:** 2024-09-25

**Authors:** Timothy J. J. Inglis

**Affiliations:** 1 Schools of Medicine and Biomedical Sciences, University of Western Australia, Crawley, Western Australia, Australia; 2 Department of Microbiology, PathWest Laboratory Medicine, Nedlands, Western Australia

**Keywords:** antimicrobial resistance, AMR, antimicrobials, clinical microbiology, KO AMR, United Nations

Few readers will have missed the lead-up to the United Nations General Assembly debate on antimicrobial resistance. Such is the importance of AMR as a real and present danger to healthcare as we know it that the issue is being raised at the highest possible international level. As our contribution to global effort, I had a note in my diary to conduct a virtual interview with the colleagues, Tina Joshi and Catrin Moore, who lead the Knock Out AMR (KO AMR) project.

## AMR has been high on the public agenda for over a decade now. What do you see KO AMR bringing to the table that was not there before?

### Tina

KO AMR is a 5-year *Microbiology Society*-driven incentive that allows microbiologists to be represented in AMR policy conversations. There are many advocates for antimicrobial resistance (AMR) action across the industry, but having subject matter experts can often provide essential scientific context to AMR when policy conversations are happening. Moreover, we have designed KO AMR to include varying stakeholder voices in an effort to identify critical gaps in addressing AMR. Through highlighting these gaps, we can start to bring together information and data to support real change at policy and scientific levels.

### Catrin

As a 5-year project, KO AMR has an ambition to pull a collective response to feed into inquiries and policies over time. KO AMR has already brought together over 200 scientific experts in the field examining four areas of AMR (surveillance, diagnostics, vaccines and therapeutics) to feed into policy using a solution-focused complex system approach. We have brought a cohort of people together who have answered an online survey that has been fed back to the UK Health Security Agency (HSA) when the new UK AMR national action plan was published. The team is agile and responsive in our approach to minimize AMR.

## Is KO AMR just for UK organizations?

### Tina

No, definitely not! It is for everyone and anyone who is interested in AMR and wants to support AMR awareness. The reach of KO AMR is already global – both I and Catrin represent the *Microbiology Society* at the UN High-Level Meeting on AMR on 26 September (2024) in New York. KO AMR is about putting unheard voices forward and trying to find solutions to, arguably, the greatest medical problem of our time. The more people behind KO AMR – the better!

### Catrin

We are committed to making sure previously unheard voices have a platform to tell their stories. Many of the team are early career researchers and/or based in the Global South and will be able to spotlight the huge challenge we have across many countries globally. Within our new *AMR in Focus* series, we are also making sure we are supporting the careers of our younger *Microbiology Society* members through training interspersed with research meetings.

## How do you see *JMM* contributors getting involved in KO AMR?

### Tina

It would be great to see more submissions to *JMM* from researchers who are actively undertaking medical and clinical research in AMR. We are planning an AMR collection and any contributions will automatically contribute to the KO AMR project and help us gather important information and evidence for policy change. Evidence and data in the form of publications can change the tide with AMR. Please submit to *JMM*, support the *Microbiology Society*, which is a not-for-profit publisher and support other researchers in the field to publish their work too!

### Catrin

If you are a *JMM* contributor doing AMR-related research, why would not you get involved in KO AMR? Collaboration from many different areas of expertise is the only way to minimize AMR. Our collective voices can be loud and make a difference in the research that is performed and in reaching out to community members and policymakers.

## If you had a seat in the World Health Assembly to represent KO AMR, what one initiative would you advocate?

### Tina

I would advocate for better use and development of diagnostic technologies to aid in surveillance of AMR. Why? Well, we have an excellent Subscription model already set up in the UK that is having an impact on prescribing, and there is a strong global incentive to invest in the R&D pipeline for antibiotics and antimicrobials. *But* if we have better diagnostics, we could better prescribe antimicrobials when required and understand the prevalence of AMR in certain populations – allowing us to better gauge the scale of AMR. This extends to being able to diagnose AMR as a cause of death and this being written as such on death certificates. It is what is called a ‘domino effect’. So, I would advocate for appropriate diagnostics, as the impact is huge.

### Catrin

I cannot argue with the need to have better diagnostics; however, linked to this, I would advocate for better quality surveillance data collection that is shared widely. This better quality data (microbiology, clinical, antimicrobial use and outcome data) will provide the evidence needed to support new interventions and policies to minimize AMR in the future. While the aspiration would be to do this using the One Health lens, we need to begin by prioritizing human health as an exemplar that can be followed for animal health and the environment.

## What would you like to see KO AMR achieve in the next decade?

### Tina

I would like to see the new gap analysis reports from our KO AMR workshops actively contribute to written evidence in policy incentives and legislation. I would like to see AMR filter into the public psyche through KO AMR activities. It would be amazing to see a world where AMR is a hot topic, such as climate change, and people on the street understand why it is important and care. If we manage to do that – we have done a lot!

### Catrin

In line with Tina, I think we need to follow the climate change lead and have an intergovernmental panel on AMR, pulling together global evidence on AMR, bringing this problem to the forefront of people’s psyches with an understanding of the problem and being discussed by the younger generation. It would be fantastic if the complex system analysis from the KO AMR workshops in January this year supports further discussion in the field and is used and expanded upon by many researchers and even used by the intergovernmental panel.

## Where can interested readers find out more about KO AMR?

### Tina

You can find out more about KO AMR via the website: Knocking Out Antimicrobial Resistance | Microbiology SocietyKnocking Out Antimicrobial Resistance | Microbiology Society [https://microbiologysociety.org/why-microbiology-matters/knocking-out-antimicrobial-resistance.html#:~:text=The%20Knocking%20Out%20AMR%20project%20aims%20to%20bring%20together%20everyone,with%20opportunities%20to%20get%20involved] and the gap analysis reports will be online soon. Check it out and get involved!

### Catrin

You can always reach out to Tina and me with any questions as well (particularly if you are in New York at the end of September); you will find us very approachable and happy to discuss AMR.

TJJI, 9-SEP-24.

